# Assessment of venous pressure by compression sonography of the internal jugular vein during 3 days of bed rest

**DOI:** 10.1113/EP091372

**Published:** 2023-10-12

**Authors:** Christopher M. Hearon, Kirsten Peters, Katrin A. Dias, James P. Macnamara, John E. T. Marshall, Joseph Campain, David Martin, Karina Marshal‐Goebel, Benjamin D. Levine

**Affiliations:** ^1^ Institute for Exercise and Environmental Medicine Texas Health Presbyterian Hospital Dallas Dallas TX USA; ^2^ University of Texas Southwestern Medical Center Dallas TX USA; ^3^ University Medical Center Radboud University Nijmegen the Netherlands; ^4^ KBR Houston TX USA; ^5^ NASA Johnson Space Center Houston TX USA

**Keywords:** central venous pressure, compression sonography, jugular vein, microgravity, spaceflight

## Abstract

Compression sonography has been proposed as a method for non‐invasive measurement of venous pressures during spaceflight, but initial reports of venous pressure measured by compression ultrasound conflict with prior reports of invasively measured central venous pressure (CVP). The aim of this study is to determine the agreement of compression sonography of the internal jugular vein (IJVP) with invasive measures of CVP over a range of pressures relevant to microgravity exposure. Ten healthy volunteers (18–55 years, five female) completed two 3‐day sessions of supine bed rest to simulate microgravity. IJVP and CVP were measured in the seated position, and in the supine position throughout 3 days of bed rest. The range of CVP recorded was in line with previous reports of CVP during changes in posture on Earth and in microgravity. The correlation between IJVP and CVP was poor when measured during spontaneous breathing (*r =* 0.29; *R* ^2^ = 0.09; *P* = 0.0002; standard error of the estimate (SEE) = 3.0 mmHg) or end‐expiration CVP (CVP_EE_; *r =* 0.19; *R* ^2^ = 0.04; *P* = 0.121; SEE = 3.0 mmHg). There was a modest correlation between the change in CVP and the change in IJVP for both spontaneous ΔCVP (*r =* 0.49; *R* ^2^ = 0.24; *P* < 0.0001) and ΔCVP_EE_ (*r =* 0.58; *R* ^2^ = 0.34; *P* < 0.0001). Bland–Altman analysis of IJVP revealed a large positive bias compared to spontaneous breathing CVP (3.6 mmHg; SD = 4.0; CV = 85%; *P* < 0.0001) and CVP_EE_ (3.6 mmHg; SD = 4.2; CV = 84%; *P* < 0.0001). Assessment of absolute IJVP via compression sonography correlated poorly with direct measurements of CVP by invasive catheterization over a range of venous pressures that are physiologically relevant to spaceflight. However, compression sonography showed modest utility for tracking changes in venous pressure over time.

## Abstract (highlights)

- **What is the central question of this study?**
  Compression sonography has been proposed as a novel method for non‐invasive measurement of venous pressures during spaceflight. However, the accuracy has not yet been confirmed in the range of CVP experienced by astronauts during spaceflight.
- **What is the main finding and its importance?**
  Our data show that compression sonography of the internal jugular vein correlates poorly with direct measurement of central venous pressures in a range that is physiologically relevant to spaceflight. However, compression sonography showed modest utility for tracking changes in venous pressure over time.

## 1 INTRODUCTION

The loss of hydrostatic gradients during exposure to microgravity results in a headward shift of blood volume and cerebral spinal fluid that contributes to key cardiovascular, cerebrovascular and ocular adaptations to spaceflight (Lawley et al., 2017; Lee et al., 2017; Norsk, 2020; Zhang & Hargens, 2014). While on Earth, approximately two‐thirds of an average day is spent seated or standing upright which lowers venous and intracranial pressure. During short‐duration exposure to microgravity, intra‐cranial and venous pressures are lower than the supine position on Earth (Buckey et al., 1993, 1996; Foldager et al., 1996; Lawley et al., 2017; Norsk et al., 1987; Videbaek & Norsk, 1997) but remain higher than the upright position (Lawley et al., 2017). Therefore, the inability to lower venous (above the heart) and intra‐cranial pressure during spaceflight results in a mild but persistent elevation in daily intra‐cranial pressure and venous pressure that may cause detrimental structural changes in the brain (Kramer et al., 2020; Roberts et al., 2017), reduced cerebral venous outflow (Marshall‐Goebel et al., 2019) and ocular remodelling (Mader et al., 2011). Therefore, technologies that measure and monitor venous pressure and haemodynamics are needed to determine the mechanisms contributing to adverse health outcomes during long‐duration spaceflight and develop potential countermeasures (Hearon et al., 2022; Marshall‐Goebel et al., 2021).

Central venous pressure (CVP) is a key haemodynamic parameter relevant to the assessment of venous compliance, cephalad fluid shift and change in cardiac filling pressure, and it has a strong linear relationship with intracranial pressure (Buckey et al., 1996; Hansen et al., 2021; Lawley et al., 2017; Mader et al., 2011; Martin et al., 2016). However, measurements of CVP are challenging. The gold‐standard technique to measure CVP is by venous catheters inserted into the central circulation that directly measure pressure in the superior vena cava near the right atrium. Unfortunately, these measurements are invasive, and resource intensive and carry a potential risk of infection, infiltration, phlebitis and thrombosis (Akmal et al., 2007; Ciozda et al., 2016; Gonzalez & Cassaro, 2021; Marsh et al., 2020; Merrer et al., 2001). Therefore, the internal jugular vein is often used as a non‐invasive surrogate for venous pressure due to its role in cerebral venous drainage and direct connection to the central venous circulation. Compression sonography has been proposed as a novel method for non‐invasive measurement of venous pressures during spaceflight (Marshall‐Goebel et al., 2019; Martin et al., 2016). In this technique, a fluid‐filled bladder and manometer is fitted to an ultrasound probe to quantify hold‐down pressure while imaging a vein. The hold‐down pressure required to collapse the vein is assumed to be equal to the pressure within the vein (Baumann et al., 2005).

Compression sonography of forearm veins has been shown to correlate well with measures of central and peripheral venous pressure when compared over a wide range of venous pressures (0–80 mmHg) induced experimentally, or in intensive care patients (Thalhammer et al., 2007; Tomoeda et al., 2020). However, the range over which CVP is measured in these studies is often substantially larger than the physiological range of CVP experienced by healthy astronauts during spaceflight (Martin et al., 2016), and data supporting the accuracy of compression sonography on larger, deeper veins in the neck are limited. A single study comparing compression ultrasound of the jugular veins to invasive measurements of CVP showed a poor correlation in the range of 0–10 mmHg (Tomoeda et al., 2020). Finally, recent reports using compression ultrasound indicate that absolute values of IJVP in microgravity and during spaceflight are substantially higher than previous reports of CVP measured invasively (Martin et al., 2016). Therefore, the aim of the current study is to determine the agreement of compression sonography of the internal jugular vein with invasive measures of CVP over a range that is relevant to microgravity exposures during 3 days’ simulated spaceflight.

## 2 METHODS

### 2.1 Ethics approval

Investigations were conducted at UT Southwestern Medical Center (Dallas, TX, USA) and the investigation was approved by the institutional review board of the University of Texas Southwestern Medical Center (STU 102015‐057) and followed guidelines set forth in the *Declaration of Helsinki* (NCT05016414) (Hearon et al., 2022). Participants provided written informed consent and were compensated for their time.

### 2.2 Overview

Investigations took place between May 2019 and February 2020 as part of a trial investigating lower body negative pressure (LBNP) as a countermeasure for the development of spaceflight associated neuro‐ocular syndrome, SANS (NCT05016414) (Hearon et al., 2022). The objective of this study was to determine whether partial reintroduction of a footward fluid shift during simulated microgravity via LBNP during sleep would attenuate choroid engorgement, which is an early marker of ocular remodelling related to SANS. The current study is a secondary analysis of the CVP and IJVP measurements made during this study.

### 2.3 Study design

Ten healthy volunteers (age 18–55 years, five female) free of pre‐existing cardiovascular, kidney, or ophthalmological disease were enrolled and completed two separate 3‐day sessions of strict supine bed rest to simulate microgravity. Each 3‐day exposure was separated by at least a 10‐day washout. In a randomized crossover design (1:1, simple randomization), participants received 8 h of overnight LBNP (−20 mmHg, 22.00–06.00 h) by a custom‐designed sleeping sack sealed at the level of the umbilicus or control conditions (no LBNP). This dose of LBNP has previously been shown to acutely reduce intracranial pressure and central venous pressure without compromising cerebral perfusion (Petersen et al., 2019). In the current study, we present IJVP and CVP measured from the seated and supine position for the duration of 3 days of supine bed rest.

### 2.4 Experimental protocol

**FIGURE 1:**
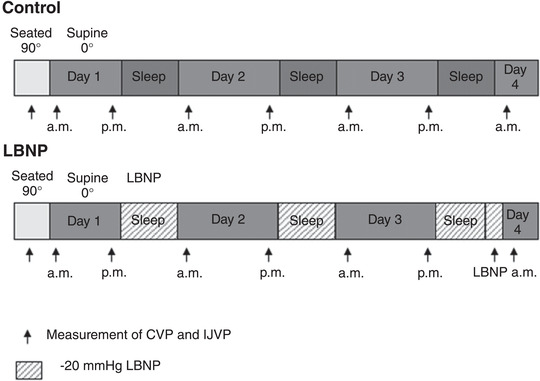
Study overview. Each participant completed two 3‐day‐long bouts of bed rest separated by a minimum of 10 days of washout. Measurements of central venous pressure (CVP) and internal jugular venous pressure (IJVP) were made in the upright position, followed by measurements in the supine position every morning (a.m.) and evening (p.m.) thereafter. During the lower body negative pressure trials (LBNP) participants slept in a specialized sack designed to deliver −20 mmHg LBNP for 8 h each night. Measurements were made at the same time points as control conditions with LBNP turned off. An additional measurement of CVP was made with LBNP turned on during the morning of Day 4 to demonstrate the acute effect of LBNP on CVP and IJVP.

On the first morning of the study, a peripherally inserted venous catheter was placed for the measurement of CVP. After 20 min of seated rest (90° upright) measurements of CVP and IJVP were acquired as described below. Participants then transitioned to the supine position and rested for an additional 20 min prior to repeating CVP and IJVP measurements. Thereafter, measurements were repeated in the supine position at 08.00 and 20.00 h during each day of bed rest. During the LBNP trials, all measurements were made in the evening prior to turning LBNP on, and in the morning after LBNP was turned off and haemodynamics were stabilized. An additional measurement of CVP and IJVP was made with LBNP still turned on during the fourth morning to quantify the acute effect of LBNP (Figure 1).

### 2.5 Measurements

#### 2.5.1 CVP

A fluid‐filled 4F catheter was placed into a brachial vein. The catheter was advanced to the level of right atrium, and the correct position was confirmed with fluoroscopy and through the identification of CVP waveforms. The position of the right atrium was marked on the body in the anteroposterior and lateral planes, and the pressure transducer (Transpac IV; ICU Medical, San Clemente, CA, USA) was levelled at the right atrium and zeroed to atmospheric pressure in all conditions (Hearon et al., 2022). During all conditions (i.e., control and LBNP) measurements of CVP were made during 5 min of spontaneous breathing followed by measurements of IJVP. In a subset of measurements made during control conditions, CVP was measured simultaneously during IJVP measurements during an end‐expiratory breath hold (CVP_EE_).

#### 2.5.2 IJVP

IJVP was measured using a custom‐made compression sonography device (VeinPress) consisting of a bladder filled with an ultrasound‐translucent mixture of water and glycerin (Baumann et al., 2005). The bladder was connected to a manometer and attached to the head of a 12 MHz linear array ultrasound probe (Vivid 7, General Electric, Milwaukee, WI, USA). The right internal jugular vein was selected for these measurements because it is superficial with no firm structures overlaying that could impede compression, and it is commonly used for assessments of venous pressure in microgravity research (Martin et al., 2015). After the supine baseline measurements, the location of placement of the ultrasound probe was marked on the neck and anatomical landmarks were identified to ensure uniform measurement location. The device was zeroed prior to compression by holding the bladder just above the skin without compressing the vein. For making a measurement, the VeinPress was slowly pressed against the skin while compressing the internal jugular vein until the point of closure as verified by ultrasound imaging. The pressure measurements were recorded a minimum of three times with a release of pressure in between. Participants were instructed to hold their breath at end‐expiration during measurements to minimize the influence of respiration on venous pressure (Martin et al., 2016). Measurements were taken by an experienced sonographer with specific training in the compression ultrasound technique who was blinded to the pressure measurement. Pressure readings were acquired online during each measurement, and videos of each measurement were recorded and stored for offline analysis. Video files were analysed offline by an independent operator experienced in the analysis and interpretation of the IJVP technique who was blinded to condition and direct CVP measurements.

### 2.6 Statistical analyses

The effect of postural changes, 3 days of bed rest, and acute LBNP on CVP and IJVP was assessed using mixed effects analysis with the Greenhouse–Geisser correction for sphericity. Post‐hoc comparisons using Dunnett's multiple comparisons tests were performed to compare each time point to the first supine measurement made on Day 1 (baseline). Simple linear regression was used to assess the relationship and standard error of the estimate (SEE) between CVP or CVP_EE_ and IJVP, as well as the change in CVP or CVP_EE_ and IJVP from baseline. Bland–Altman plots were used to determine the bias and agreement of the two methods in measuring absolute venous pressure as well as changes in pressure over time. Mean bias was evaluated using a one sample Student's *t*‐test versus a null hypothesis of 0 mmHg. Proportional bias was assessed by linear regression of the difference between methods versus the average of the two methods. The coefficient of variation was calculated as SD of bias divided by criterion mean. A value of *P* < 0.05 was considered statistically significant. Continuous variables are expressed as means ± SD or 95% confidence interval (CI), categorical variables are expressed as *n* (%). Statistical analysis was performed using GraphPad Prism 9.4.1 (GraphPad Software, Boston, MA, USA).

## 3 RESULTS

Participant characteristics at baseline are provided in Table 1. Out of 170 potential paired measurements of IJVP and CVP during spontaneous breathing, 156 were used in the final analysis. Out of the 80 potential paired simultaneous measurements of IJVP and CVP during end‐expiratory breath hold, 69 were used in the final analysis. Two spontaneous breathing CVP measurements (<2%) and six CVP_EE_ measurements (<10%) were not made, and 13 IJVP measurements (<10%) were either not made or not sufficient for video analysis.

**TABLE 1:** Baseline characteristics.

Characteristic	Value
Age (years)	29 (9)
Sex, female (*n* (%))	5 (50)
Height (cm)	172 (11.5)
Weight (kg)	68.4 (10.4)
BMI (kg/m^2^)	23.1 (1.5)
SBP (mmHg)	117 (12)
DBP (mmHg)	76 (10)
Heart rate (bpm)	68 (10)
Supine CVP (mmHg)	6.8 (1.6)

Data are means (SD) except where stated otherwise. Abbreviations: CVP: central venous pressure; DBP: diastolic blood pressure; SBP: systolic blood pressure.

### 3.1 Effect of posture, bedrest and acute LBNP on CVP and IJVP

**FIGURE 2:**
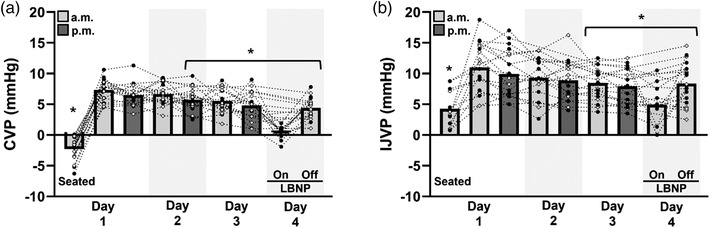
CVP and IJVP during 3 nights of bed rest. (a) Central venous pressure (CVP) during spontaneous breathing and (b) Internal jugular venous pressure (IJVP) measured first in the seated position, and then supine during each morning (a.m.) and evening (p.m.) of bed rest. Open circles indicate control trials, filled circles indicate trials that included 8 h of −20 mmHg lower body negative pressure (LBNP) each night. An additional measurement of CVP and IJVP was made on Day 4 a.m. with LBNP on. *P < 0.05 vs. Day 1 a.m. supine.

Moving from the seated to the supine position increased both CVP (+9.6 mmHg; 95% CI: 8.4 to 10.8 mmHg) and IJVP (+6.7 mmHg; 95% CI: 2.7 to 10.8 mmHg). Over the course of 3 days of bedrest there was a gradual decline in CVP (−2.9 mmHg; 95% CI: −4.3 to −1.5 mmHg) and IJVP (−2.6 mmHg; 95% CI: −0.3 to 5.0 mmHg) from the morning measurement on Day 1 to the morning measurement on Day 4, and acute LBNP was effective in lowering both CVP (−7.1 mmHg; 95% CI: −9.0 to −5.1 mmHg) and IJVP (−6.1 mmHg; 95% CI: −11.4 to −0.7 mmHg). A decline in CVP of −1.6 mmHg (95% CI: −3.1 to −0.1 mmHg) compared to supine baseline was detectible statistically by venous catheter at the Day 2 evening measurement, whereas a decline in IJVP of −2.5 mmHg (95% CI: −4.2 to −0.8) was detectible by Day 3 morning (Figure 2).

### 3.2 Correlation between CVP and IJVP

**FIGURE 3:**
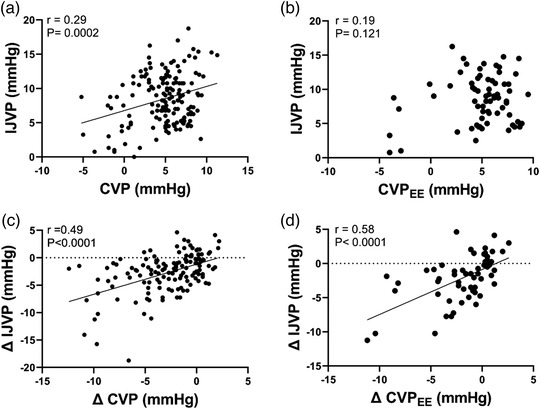
Correlation between CVP and IJVP measurements. (a,b) Correlational analysis of internal jugular venous pressure (IJVP) and (a) central venous pressure (CVP) during spontaneous breathing and (b) CVP during end expiratory breath hold (CVP_EE_). (c,d) Correlational analysis of the change in IJVP from baseline (Day 1 a.m. supine) and (c) the change in CVP during spontaneous breathing and (d) the change in CVP_EE_ from baseline.

Within the physiological range of CVP recorded in this investigation (−5 to 10 mmHg) the correlation between IJVP and CVP was poor during both spontaneous breathing CVP (*r =* 0.29; *R* ^2^ = 0.09; *P* = 0.0002; SEE = 3.0) and simultaneous CVP_EE_ (*r =* 0.19; *R* ^2^ = 0.04; *P* = 0.121; SEE = 3.0) (Figure 3a,b). However, there was a modest correlation between the change in CVP and the change in IJVP for both spontaneous breathing ΔCVP (*r =* 0.49; *R* ^2^ = 0.24; *P* < 0.0001) and ΔCVP_EE_ (*r =* 0.58; *R* ^2^ = 0.34; *P* < 0.0001) (Figure 3c,d).

### 3.3 Bland–Altman analysis

**FIGURE 4:**
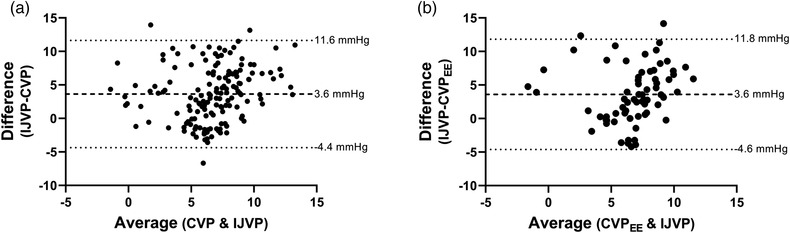
Bland–Altman analysis of CVP and IJVP. Bland–Altman analysis of internal jugular venous pressure (IJVP) and (a) central venous pressure (CVP) during spontaneous breathing and (b) CVP during end expiratory breath hold (CVP_EE_). Mean bias (dashed line) and 95% limits of agreement (dotted lines) are presented. The analysis shows a fixed error of 3.6 mmHg when comparing IJVP versus both spontaneous breathing CVP and CVP_EE_.

Bland–Altman analysis of IJVP versus spontaneous breathing CVP indicated the presence of a fixed error compared to spontaneous breathing CVP (3.6 mmHg; SD 4.0; CV = 85%; *P* < 0.0001) and CVP_EE_ (3.6 mmHg; SD 4.2; CV = 84%; *P* < 0.0001) (Figure 4). Proportional bias was low for IJVP compared to spontaneous breathing CVP (*R* ^2^ = 0.09) and CVP_EE_ (*R* ^2^ = 0.04). The 95% limits of agreement for IJVP and spontaneous breathing CVP were −4.4 to 11.6 mmHg, and −4.6 to 11.8 mmHg for CVP_EE_.

## 4 DISCUSSION

The primary findings of this investigation are that within the physiological range of central venous pressures experienced during routine postural changes and expected to be encountered during exposure to microgravity, venous pressure measurements by compression sonography of the internal jugular vein correlated poorly with direct measurements of venous pressure by gold‐standard invasive central catheterization. However, compression sonography was modestly better at tracking changes in pressure over time. There was a positive bias indicating a tendency for IJVP to overestimate central venous pressure by approximately 4 mmHg, an error that is approximately 60% of the magnitude of baseline central venous pressure measured by catheter in this study. Therefore, these findings indicate that compression ultrasound is not accurate for determination of absolute venous pressures during spaceflight, though it may hold some utility for tracking changes in venous pressure in certain contexts compared to an appropriate baseline reference.

### 4.1 Compression sonography for measuring venous pressures

Compression sonography of forearm vessels has shown potential clinical utility as an indirect assessment of venous pressure in initial proof‐of‐concept studies (Thalhammer et al., 2007, 2009; Tomoeda et al., 2020; Uthoff et al., 2012). Notably, Thalhammer and colleagues demonstrated strong correlations between compression ultrasound of the cephalic vein and invasively measured venous pressure in healthy patients with experimentally induced venous hypertension (*r =* 0.95; range: 11–75 mmHg) and in intensive care patients (*r =* 0.84; range: 5–27 mmHg) (Thalhammer et al., 2007). However, subsequent feasibility studies incorporating multiple operators showed lower but significant *r* values of 0.58–0.68 over a range of pressures: 3–26 mmHg (Thalhammer et al., 2009); other independent investigator groups have shown somewhat lower accuracy of compression ultrasound of the forearm for assessment of CVP in intensive care patients (Spearman's correlation coefficient 0.48; range: 2–22 mmHg), with moderate utility for categorizing patients broadly into low or high CVP bins (Uthoff et al., 2012). While these data provide some evidence for the utility of compression ultrasound of forearm vessels in clinical settings when CVP may be elevated (Gutwein & Thalhammer, 2022), limited data are available evaluating the use of compression ultrasound on the larger jugular veins in healthy individuals.

A single investigation of 14 patients in an intensive care unit demonstrated a significant correlation between compression ultrasound of the external jugular vein and invasive CVP (*R* ^2^ = 0.77; range 0–17 mmHg) (Tomoeda et al., 2020). Here we show much lower correlations for absolute pressure values measured during spontaneous breathing (*r =* 0.30; *R* ^2^ = 0.09; range −5 to 12 mmHg). One important difference between our study and this previous study is the incorporation of postural changes, which in the upright position generates some negative CVP values due to respiratory excursions. As a result of the nature of compression sonography it is not possible to quantify negative pressures or low pressures that collapse veins, though exclusion of these values did not improve the correlation between the two measurements (*r =* 0.13). Most importantly, while our absolute range is similar to that of Tomoeda et al., all but two of our measurements occurred at central venous pressures <10 mmHg, a value below which many investigations have not measured (Thalhammer et al., 2007); when made in this range most investigations show weak correlation between compression sonography and CVP (Martin et al., 2015; Thalhammer et al., 2009; Tomoeda et al., 2020). Therefore, in situations where the range of CVP is not expected to be above 10 mmHg or pathologically high, compression sonography is not suitable for measuring venous pressures, but may hold modest utility for tracking changes in venous pressures during the course of an exposure or intervention.

### 4.2 Measuring CVP and IJVP in space

Few investigations have directly measured CVP during exposure to microgravity. However, the limited data available are remarkably consistent and indicate that CVP is lower in microgravity than in the supine position on Earth. Of the studies that have used central venous catheters to measure CVP in the supine position vs. microgravity, all report supine CVP to be in the normal range of 6–8 mmHg (Buckey et al., 1996; Foldager et al., 1996; Norsk et al., 1987; Videbaek & Norsk, 1997), while exposure to parabolic flight lowers CVP to the range of 4–7 mmHg (Foldager et al., 1996; Norsk et al., 1987; Videbaek & Norsk, 1997), and similarly during spaceflight (*n* = 4, up to 10 h) CVP is reported to be between 2 and 6 mmHg (Buckey et al., 1996; Foldager et al., 1996). However, data using compression sonography to measure internal jugular venous pressure, which should be similar to CVP during microgravity since the jugular veins are fully distended and open to the central circulation, are less consistent and at times opposite in direction to catheter‐based measurements of CVP and other peripheral venous circulations of the upper body (Kirsch et al., 1984). The most comprehensive study of compression sonography in microgravity induced by parabolic flight showed a 1.5‐fold increase in internal jugular venous pressure (from ∼10 to ∼24 mmHg), with a range of ∼20 mmHg (Martin et al., 2016). An investigation during spaceflight (50 and 150 days) showed a smaller and more variable increase in IJVP by compression sonography at 50 days compared to the supine position (supine: 17 vs. Day 50: 21 mmHg), followed by a return of IJVP pressures to levels similar to, or below the supine position in 1G (Day 150: 16 mmHg). While the temporal pattern of IJVP measured by compression sonography during spaceflight is more in‐line with catheter‐based measurements of changes in central venous pressure when compared to the supine position, the absolute values are considerably higher and generally non‐physiological (range 9–30 mmHg). This error has important implications for the study of jugular venous flow and cerebral drainage given recent reports of jugular venous flow stasis and thrombosis during spaceflight (Marshall‐Goebel et al., 2019). If the absolute IJVPs measured with compression sonography during space flight were accurate, the existence of such large pressure gradients from the internal jugular vein to the vena cava would be incompatible with the observation of venous stasis and retrograde flow, barring an obstruction of venous outflow from the internal jugular veins just cephalad of the right atrium. It is uncertain how such an obstruction could routinely occur and therefore we conclude that absolute measures of IJVP performed using compression ultrasonography in space may be quantitatively inaccurate.

One more likely explanation for the discrepancy between invasive pressure measurements and compression sonography is the loss of tissue compressive forces and alterations in transmural pressure across the veins and soft tissue of the neck during microgravity exposure. During Earth‐based studies, interventions that increase intravascular pressure have little effect on extravascular pressure. Therefore, the extramural pressure applied by the ultrasound probe will equal the change in intravascular pressure. However, during spaceflight both intravascular pressure and extravascular pressure change such that transmural pressure in some instances is maintained or increased (Buckey et al., 1996). Therefore, the seeming overestimation of IJVP compared to invasive CVP could be due to the effects of reduced extramural pressure secondary to the loss of tissue compressive forces of gravity. In this scenario, the pressure measured by compression sonography, which modulates extramural pressure to compress veins, could be overestimated. It also might require more external hold‐down pressure to compress the jugular veins based on their changes in shape and alterations in the surrounding tissue pressure. Especially during parabolic flight when the operator has only a few seconds to make the measurements in a moving plane, the technique may be further compromised. Therefore, compression sonography may be more useful for tracking changes in intravascular pressure over time, or for assessing the effectiveness of countermeasures designed to reduce venous pressure and increase venous drainage during spaceflight, only when compared to a baseline condition with similar tissue compressive forces (i.e., a 0G baseline).

### 4.3 Considerations

There are several considerations to take into account regarding our study. First, the pressure range included in this investigation (−5 to 10 mmHg) is relatively small compared to previous investigations but is inclusive of the central venous pressures reported during spaceflight. There are limited data on venous pressures during spaceflight and as such there may be greater variability of central venous pressures during spaceflight than has previously been reported. Therefore, conclusions related to the performance of compression sonography are limited to the range of pressures reported here. Second, the use of bed rest as a spaceflight analogue does not fully reproduce the physiological stimulus of microgravity, specifically the loss of tissue compressive forces.

### 4.4 Conclusions

Compression sonography of the internal jugular vein correlated poorly with direct measurements of central venous pressure by invasive catheterization over a range of venous pressures that are physiologically relevant to spaceflight with a very large error. However, compression sonography was modestly better at tracking changes in pressure over time. Importantly, changes in transmural pressure that occur in microgravity due to the loss of tissue compressive forces reduce the utility of compression sonography for accurately assessing IJVP in space. However, when compared to an appropriate baseline measurement made in microgravity, this technique may be useful for tracking changes in venous pressure and help quantify the mechanisms that govern cerebral venous outflow during long duration spaceflight.

## AUTHOR CONTRIBUTIONS

Christopher M. Hearon Jr.—conception and design of the work, acquisition, analysis and interpretation of data, drafting the work. Kirsten Peters—acquisition, analysis and interpretation of data, drafting the work. Katrin A. Dias—conception and design of the work, acquisition, analysis and interpretation of data, drafting the work. James P. Macnamara—acquisition, analysis and interpretation of data. John E.T. Marshall—analysis and interpretation of data. Joseph Campain—acquisition, analysis and interpretation of data. David Martin—analysis and interpretation of data. Karina Marshal‐Goebel—interpretation of data. Benjamin D. Levine—conception and design of the work, analysis and interpretation of data, drafting the work. All authors have read and approved the final version of this manuscript and agree to be accountable for all aspects of the work in ensuring that questions related to the accuracy or integrity of any part of the work are appropriately investigated and resolved. All persons designated as authors qualify for authorship, and all those who qualify for authorship are listed.

## CONFLICT OF INTEREST

The authors declare no conflicts of interest.

## Data Availability

Data supporting the findings of this study are available from the corresponding author upon reasonable request.

## References

[eph13434-bib-0001] Akmal, A. H. , Hasan, M. , & Mariam, A. (2007). The incidence of complications of central venous catheters at an intensive care unit. Annals of Thoracic Medicine, 2(2), 61–63. 19727348 10.4103/1817-1737.32232 PMC2732078

[eph13434-bib-0002] Baumann, U. A. , Marquis, C. , Stoupis, C. , Willenberg, T. A. , Takala, J. , & Jakob, S. M. (2005). Estimation of central venous pressure by ultrasound. Resuscitation, 64(2), 193–199. 15680529 10.1016/j.resuscitation.2004.08.015

[eph13434-bib-0003] Buckey, J. C. , Gaffney, F. A. , Lane, L. D. , Levine, B. D. , Watenpaugh, D. E. , & Blomqvist, C. G. (1993). Central venous pressure in space. New England Journal of Medicine, 328(25), 1853–1854. 8502279 10.1056/NEJM199306243282516

[eph13434-bib-0004] Buckey, J. C., Jr. , Gaffney, F. A. , Lane, L. D. , Levine, B. D. , Watenpaugh, D. E. , Wright, S. J. , Yancy, C. W., Jr , Meyer, D. M. , & Blomqvist, C. G. (1996). Central venous pressure in space. Journal of Applied Physiology, 81(1), 19–25. 8828643 10.1152/jappl.1996.81.1.19

[eph13434-bib-0005] Ciozda, W. , Kedan, I. , Kehl, D. W. , Zimmer, R. , Khandwalla, R. , & Kimchi, A. (2016). The efficacy of sonographic measurement of inferior vena cava diameter as an estimate of central venous pressure. Cardiovascular ultrasound, 14(1), 33. 27542597 10.1186/s12947-016-0076-1 PMC4992235

[eph13434-bib-0006] Foldager, N. , Andersen, T. A. , Jessen, F. B. , Ellegaard, P. , Stadeager, C. , Videbaek, R. , & Norsk, P. (1996). Central venous pressure in humans during microgravity. Journal of Applied Physiology, 81(1), 408–412. 8828692 10.1152/jappl.1996.81.1.408

[eph13434-bib-0007] Gonzalez, R. , & Cassaro, S. (2021). Percutaneous Central Catheter . StatPearls. 29083596

[eph13434-bib-0008] Gutwein, A. , & Thalhammer, C. (2022). Ultrasound‐guided venous pressure measurement. Vasa, 51(6), 333–340. 36200379 10.1024/0301-1526/a001032

[eph13434-bib-0009] Hansen, A. B. , Lawley, J. S. , Rickards, C. A. , Howden, E. J. , Sarma, S. , Cornwell, W. K., 3rd , Amin, S. B. , Mugele, H. , Marume, K. , Possnig, C. , Whitworth, L. A. , Williams, M. A. , & Levine, B. D. (2021). Reducing intracranial pressure by reducing central venous pressure: Assessment of potential countermeasures to spaceflight‐associated neuro‐ocular syndrome. Journal of Applied Physiology, 130(2), 283–289. 33270516 10.1152/japplphysiol.00786.2020

[eph13434-bib-0010] Hearon, C. M., Jr. , Dias, K. A. , Babu, G. , Marshall, J. E. T. , Leidner, J. , Peters, K. , Silva, E. , MacNamara, J. P. , Campain, J. , & Levine, B. D. (2022). Effect of nightly lower body negative pressure on choroid engorgement in a model of spaceflight‐associated neuro‐ocular syndrome: A randomized crossover trial. JAMA Ophthalmology, 140(1), 59–65. 34882176 10.1001/jamaophthalmol.2021.5200 PMC8662537

[eph13434-bib-0011] Kirsch, K. A. , Röcker, L. , Gauer, O. H. , Krause, R. , Leach, C. , Wicke, H. J. , & Landry, R. (1984). Venous pressure in man during weightlessness. Science, 225(4658), 218–219. 6729478 10.1126/science.6729478

[eph13434-bib-0012] Kramer, L. A. , Hasan, K. M. , Stenger, M. B. , Sargsyan, A. , Laurie, S. S. , Otto, C. , Ploutz‐Snyder, R. J. , Marshall‐Goebel, K. , Riascos, R. F. , & Macias, B. R. (2020). Intracranial effects of microgravity: A prospective longitudinal MRI Study. Radiology, 295(3), 640–648. 32286194 10.1148/radiol.2020191413

[eph13434-bib-0013] Lawley, J. S. , Petersen, L. G. , Howden, E. J. , Sarma, S. , Cornwell, W. K. , Zhang, R. , Whitworth, L. A. , Williams, M. A. , & Levine, B. D. (2017). Effect of gravity and microgravity on intracranial pressure. The Journal of Physiology, 595(6), 2115–2127. 28092926 10.1113/JP273557 PMC5350445

[eph13434-bib-0014] Lee, A. G. , Mader, T. H. , Gibson, C. R. , & Tarver, W. (2017). Space Flight‐Associated Neuro‐ocular Syndrome. JAMA Ophthalmology, 135(9), 992–994. 28727859 10.1001/jamaophthalmol.2017.2396

[eph13434-bib-0015] Mader, T. H. , Gibson, C. R. , Pass, A. F. , Kramer, L. A. , Lee, A. G. , Fogarty, J. , Tarver, W. J. , Dervay, J. P. , Hamilton, D. R. , Sargsyan, A. , Phillips, J. L. , Tran, D. , Lipsky, W. , Choi, J. , Stern, C. , Kuyumjian, R. , & Polk, J. D. (2011). Optic disc edema, globe flattening, choroidal folds, and hyperopic shifts observed in astronauts after long‐duration space flight. Ophthalmology, 118(10), 2058–2069. 21849212 10.1016/j.ophtha.2011.06.021

[eph13434-bib-0016] Marsh, N. , Webster, J. , Ullman, A. J. , Mihala, G. , Cooke, M. , Chopra, V. , & Rickard, C. M. (2020). Peripheral intravenous catheter non‐infectious complications in adults: A systematic review and meta‐analysis. Journal of Advanced Nursing, 76(12), 3346–3362. 33016412 10.1111/jan.14565

[eph13434-bib-0017] Marshall‐Goebel, K. , Laurie, S. S. , Alferova, I. V. , Arbeille, P. , Auñón‐Chancellor, S. M. , Ebert, D. J. , Lee, S. M. C. , Macias, B. R. , Martin, D. S. , Pattarini, J. M. , Ploutz‐Snyder, R. , Ribeiro, L. C. , Tarver, W. J. , Dulchavsky, S. A. , Hargens, A. R. , & Stenger, M. B. (2019). Assessment of jugular venous blood flow stasis and thrombosis during spaceflight. JAMA Network Open, 2(11), e1915011. 31722025 10.1001/jamanetworkopen.2019.15011 PMC6902784

[eph13434-bib-0018] Marshall‐Goebel, K. , Macias, B. R. , Laurie, S. S. , Lee, S. M. C. , Ebert, D. J. , Kemp, D. T. , Miller, A. , Greenwald, S. H. , Martin, D. S. , Young, M. , Hargens, A. R. , Levine, B. D. , & Stenger, M. B. (2021). Mechanical countermeasures to headward fluid shifts. Journal of Applied Physiology, 130(6), 1766–1777. 33856253 10.1152/japplphysiol.00863.2020

[eph13434-bib-0019] Martin, D. S. , Lee, S. , Stein, S. P. , Stenger, M. B. , Feiveson, A. H. , Matz, T. P. , Caine, T. L. , Scott, J. M. , Westby, C. M. , & Platts, S. H. (2015). Pilot study to evaluate a novel non‐invasive technology to measure peripheral venous pressure. *NASA/TM‐2015‐218572. NASA Johnson SpaceCenter, Houston, TX*.

[eph13434-bib-0020] Martin, D. S. , Lee, S. M. , Matz, T. P. , Westby, C. M. , Scott, J. M. , Stenger, M. B. , & Platts, S. H. (2016). Internal jugular pressure increases during parabolic flight. Physiological Reports, 4(24), e13068. 28039409 10.14814/phy2.13068 PMC5210371

[eph13434-bib-0021] Merrer, J. , De Jonghe, B. , Golliot, F. , Lefrant, J. Y. , Raffy, B. , Barre, E. , Rigaud, J. P. , Casciani, D. , Misset, B. , Bosquet, C. , Outin, H. , Brun‐Buisson, C. , & Nitenberg, G. , & French Catheter Study Group in Intensive Care . (2001). Complications of femoral and subclavian venous catheterization in critically ill patients: A randomized controlled trial. Jama, 286(6), 700–707. 11495620 10.1001/jama.286.6.700

[eph13434-bib-0022] Norsk, P. (2020). Adaptation of the cardiovascular system to weightlessness: Surprises, paradoxes and implications for deep space missions. Acta Physiologica, 228(3), e13434. 31872965 10.1111/apha.13434

[eph13434-bib-0023] Norsk, P. , Foldager, N. , Bonde‐Petersen, F. , Elmann‐Larsen, B. , & Johansen, T. S. (1987). Central venous pressure in humans during short periods of weightlessness. Journal of Applied Physiology, 63(6), 2433–2437. 3436875 10.1152/jappl.1987.63.6.2433

[eph13434-bib-0024] Petersen, L. G. , Lawley, J. S. , Lilja‐Cyron, A. , Petersen, J. C. G. , Howden, E. J. , Sarma, S. , Cornwell, W. K., 3rd , Zhang, R. , Whitworth, L. A. , Williams, M. A. , Juhler, M. , & Levine, B. D. (2019). Lower body negative pressure to safely reduce intracranial pressure. The Journal of Physiology, 597(1), 237–248. 30286250 10.1113/JP276557 PMC6312426

[eph13434-bib-0025] Roberts, D. R. , Albrecht, M. H. , Collins, H. R. , Asemani, D. , Chatterjee, A. R. , Spampinato, M. V. , Zhu, X. , Chimowitz, M. I. , & Antonucci, M. U. (2017). Effects of spaceflight on astronaut brain structure as indicated on MRI. New England Journal of Medicine, 377(18), 1746–1753. 29091569 10.1056/NEJMoa1705129

[eph13434-bib-0026] Thalhammer, C. , Aschwanden, M. , Odermatt, A. , Baumann, U. A. , Imfeld, S. , Bilecen, D. , Marsch, S. C. , & Jaeger, K. A. (2007). Noninvasive central venous pressure measurement by controlled compression sonography at the forearm. Journal of the American College of Cardiology, 50(16), 1584–1589. 17936158 10.1016/j.jacc.2007.07.022

[eph13434-bib-0027] Thalhammer, C. , Siegemund, M. , Aschwanden, M. , Gassmann, M. , Baumann, U. A. , Jaeger, K. A. , & Imfeld, S. (2009). Non‐invasive central venous pressure measurement by compression ultrasound–A step into real life. Resuscitation, 80(10), 1130–1136. 19632026 10.1016/j.resuscitation.2009.06.027

[eph13434-bib-0028] Tomoeda, H. , Sawada, K. , & Chihara, S. (2020). Noninvasive Technique for Measuring Central Venous and Arterial Pressure Using Controlled Compression Sonography. Annals of Vascular Diseases, 13(4), 397–403. 33391557 10.3400/avd.oa.20-00058 PMC7758583

[eph13434-bib-0029] Uthoff, H. , Siegemund, M. , Aschwanden, M. , Hunziker, L. , Fabbro, T. , Baumann, U. , Jaeger, K. A. , Imfeld, S. , & Staub, D. (2012). Prospective comparison of noninvasive, bedside ultrasound methods for assessing central venous pressure. Ultraschall in Der Medizin, 33(7), E256–e262. 22660962 10.1055/s-0031-1299506

[eph13434-bib-0030] Videbaek, R. , & Norsk, P. (1997). Atrial distension in humans during microgravity induced by parabolic flights. Journal of Applied Physiology, 83(6), 1862–1866. 9390956 10.1152/jappl.1997.83.6.1862

[eph13434-bib-0031] Zhang, L. F. , & Hargens, A. R. (2014). Intraocular/Intracranial pressure mismatch hypothesis for visual impairment syndrome in space. Aviation Space and Environmental Medicine, 85(1), 78–80. 24479265 10.3357/asem.3789.2014

